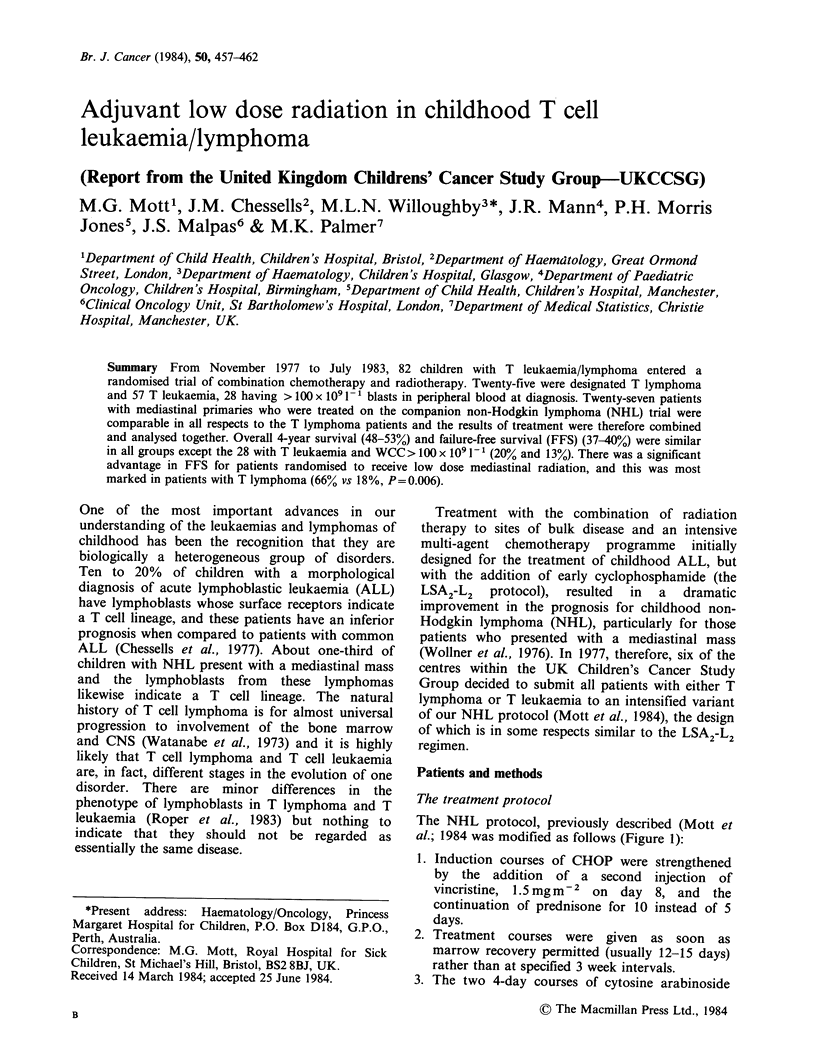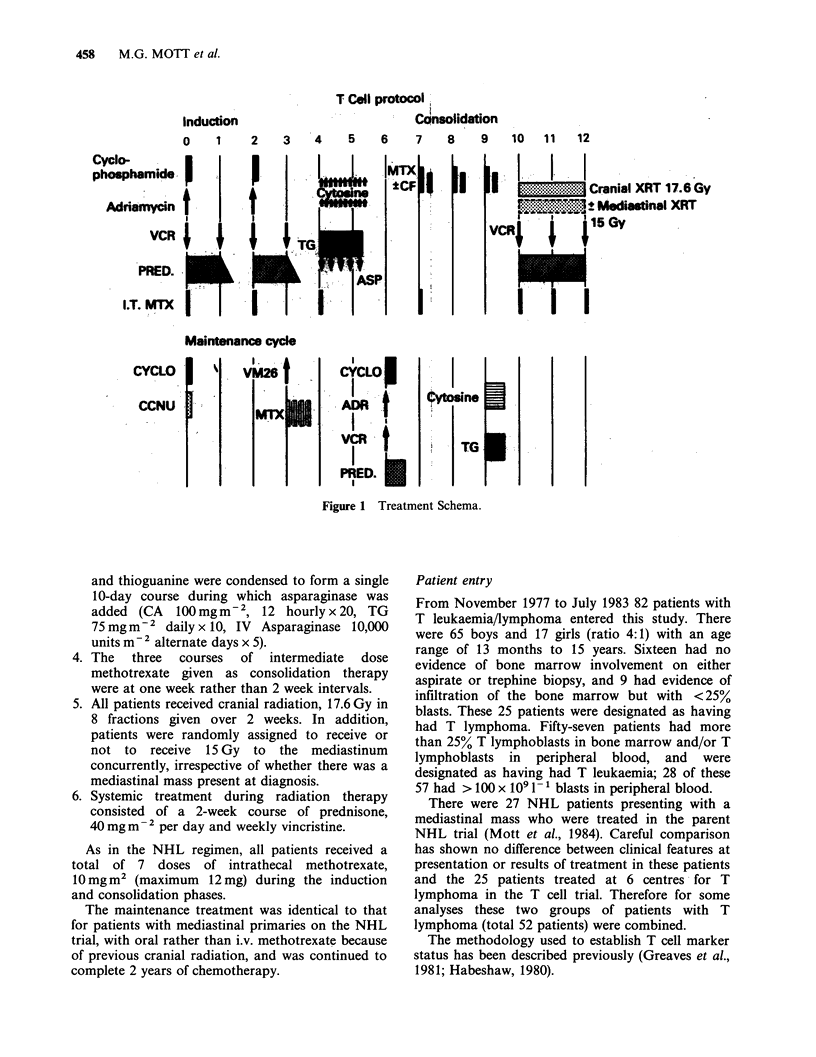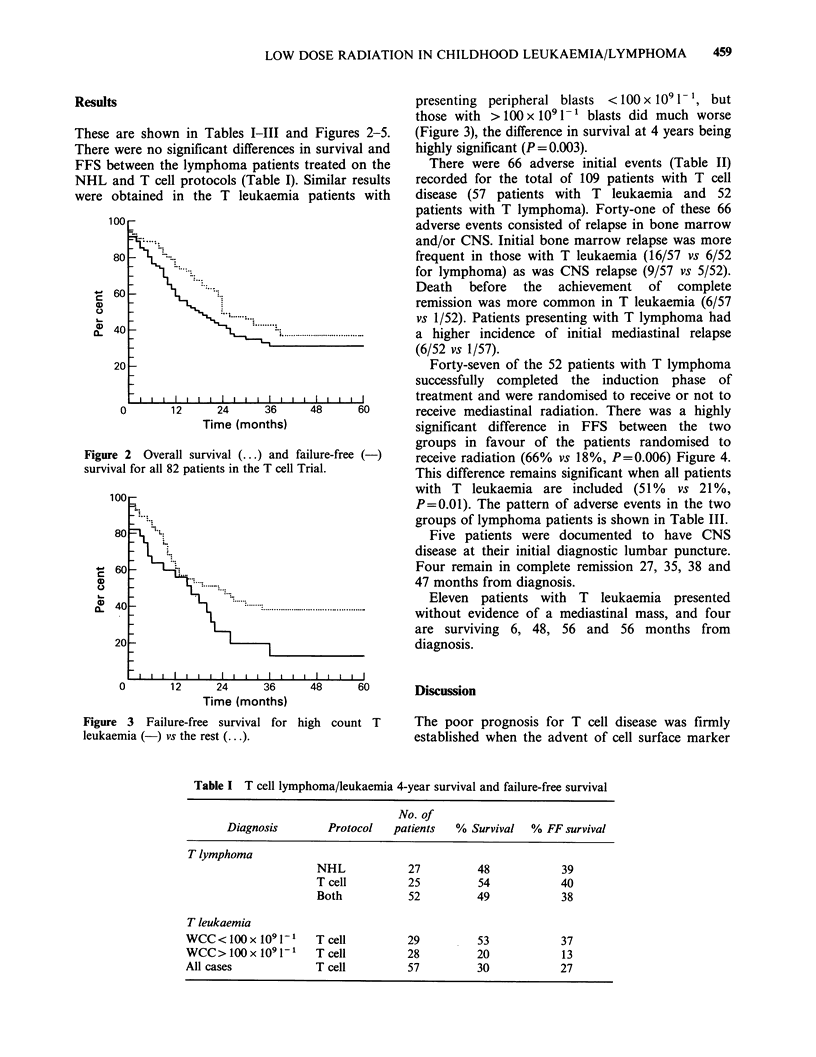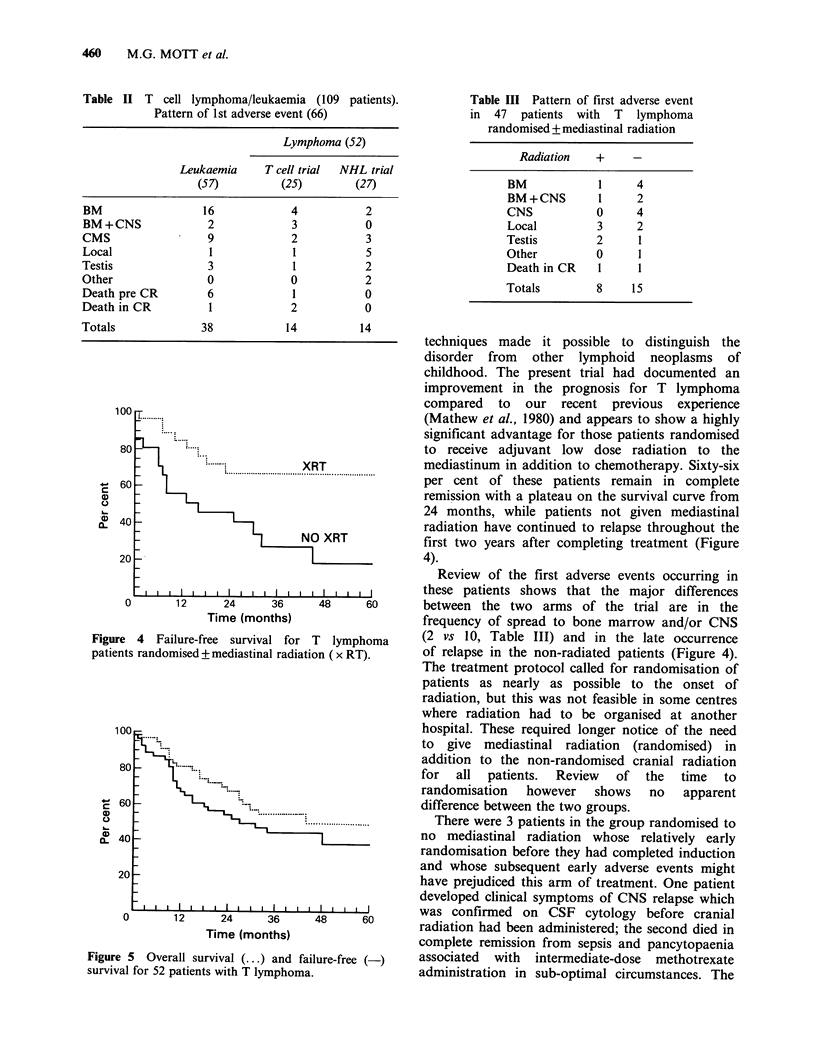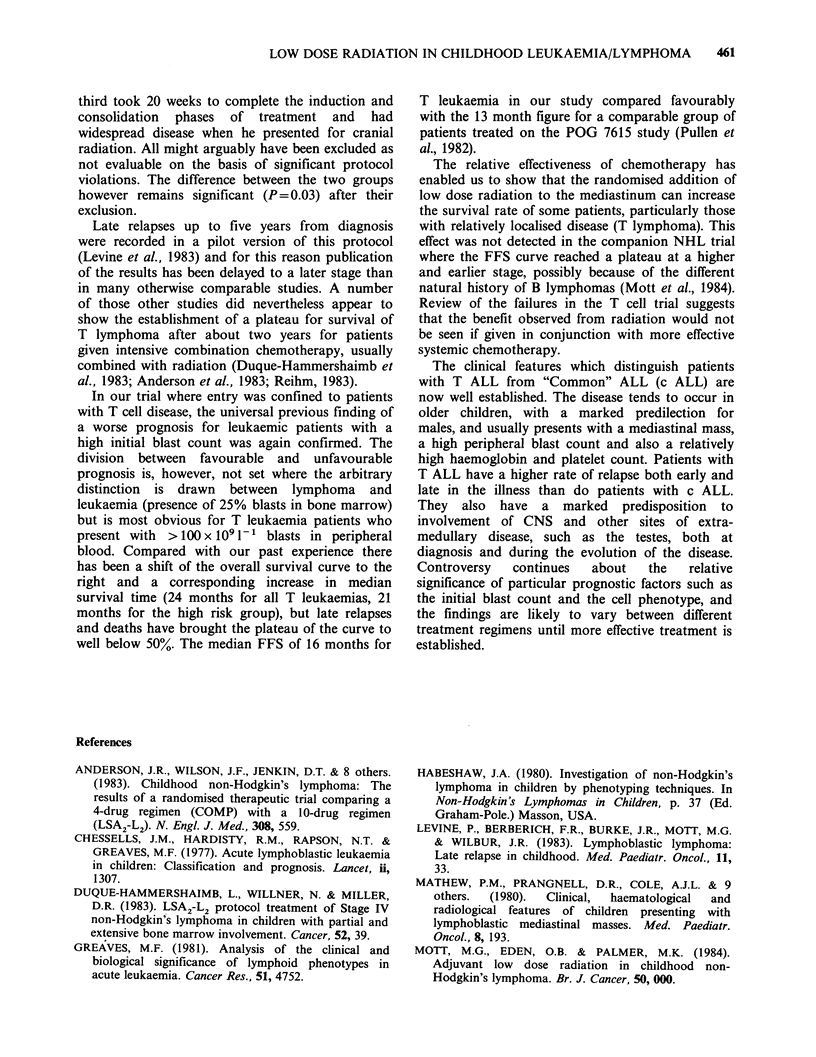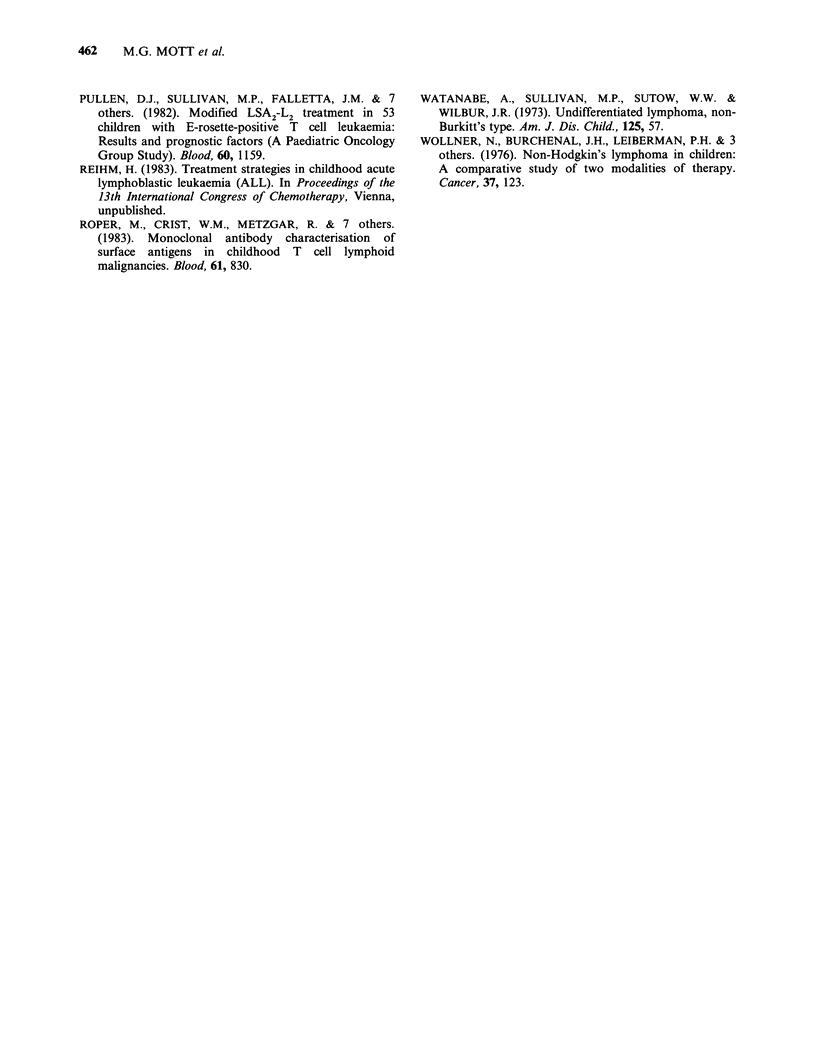# Adjuvant low dose radiation in childhood T cell leukaemia/lymphoma (report from the United Kingdom Childrens' Cancer Study Group--UKCCSG).

**DOI:** 10.1038/bjc.1984.201

**Published:** 1984-10

**Authors:** M. G. Mott, J. M. Chessells, M. L. Willoughby, J. R. Mann, P. H. Morris-Jones, J. S. Malpas, M. K. Palmer

## Abstract

From November 1977 to July 1983, 82 children with T leukaemia/lymphoma entered a randomised trial of combination chemotherapy and radiotherapy. Twenty-five were designated T lymphoma and 57 T leukaemia, 28 having greater than 100 x 10(9)1(-1) blasts in peripheral blood at diagnosis. Twenty-seven patients with mediastinal primaries who were treated on the companion non-Hodgkin lymphoma (NHL) trial were comparable in all respects to the T lymphoma patients and the results of treatment were therefore combined and analysed together. Overall 4-year survival (48-53%) and failure-free survival (FFS) (37-40%) were similar in all groups except the 28 with T leukaemia and WCC greater than 100 X 10(9)1(-1) (20% and 13%). There was a significant advantage in FFS for patients randomised to receive low dose mediastinal radiation, and this was most marked in patients with T lymphoma (66% vs 18%, P = 0.006).


*Br. J. Cancer* (1984), **50**, 457–462

### Adjuvant low dose radiation in childhood T cell leukaemia/lymphoma

### (Report from the United Kingdom Childrens' Cancer Study Group—UKCCSG)

M.G. Mott[1], J.M. Chessells[2], M.L.N. Willoughby[3]*, J.R. Mann[4], P.H. Morris Jones[5], J.S. Malpas[6] & M.K. Palmer[7]

[1]*Department of Child Health, Children's Hospital, Bristol,* [2]*Department of Haematology, Great Ormond Street, London,* [3]*Department of Haematology, Children's Hospital, Glasgow,* [4]*Department of Paediatric Oncology, Children's Hospital, Birmingham,* [5]*Department of Child Health, Children's Hospital, Manchester,* [6]*Clinical Oncology Unit, St Bartholomew's Hospital, London,* [7]*Department of Medical Statistics, Christie Hospital, Manchester, UK.*

**Summary** From November 1977 to July 1983, 82 children with T leukaemia/lymphoma entered a randomised trial of combination chemotherapy and radiotherapy. Twenty-five were designated T lymphoma and 57 T leukaemia, 28 having $>100\times10^9\,l^{-1}$ blasts in peripheral blood at diagnosis. Twenty-seven patients with mediastinal primaries who were treated on the companion non-Hodgkin lymphoma (NHL) trial were comparable in all respects to the T lymphoma patients and the results of treatment were therefore combined and analysed together. Overall 4-year survival (48–53%) and failure-free survival (FFS) (37–40%) were similar in all groups except the 28 with T leukaemia and $WCC>100\times10^9\,l^{-1}$ (20% and 13%). There was a significant advantage in FFS for patients randomised to receive low dose mediastinal radiation, and this was most marked in patients with T lymphoma (66% *vs* 18%, $P=0.006$).

One of the most important advances in our understanding of the leukaemias and lymphomas of childhood has been the recognition that they are biologically a heterogeneous group of disorders. Ten to 20% of children with a morphological diagnosis of acute lymphoblastic leukaemia (ALL) have lymphoblasts whose surface receptors indicate a T cell lineage, and these patients have an inferior prognosis when compared to patients with common ALL (Chessells *et al.*, 1977). About one-third of children with NHL present with a mediastinal mass and the lymphoblasts from these lymphomas likewise indicate a T cell lineage. The natural history of T cell lymphoma is for almost universal progression to involvement of the bone marrow and CNS (Watanabe *et al.*, 1973) and it is highly likely that T cell lymphoma and T cell leukaemia are, in fact, different stages in the evolution of one disorder. There are minor differences in the phenotype of lymphoblasts in T lymphoma and T leukaemia (Roper *et al.*, 1983) but nothing to indicate that they should not be regarded as essentially the same disease.

*Present address: Haematology/Oncology, Princess Margaret Hospital for Children, P.O. Box D184, G.P.O., Perth, Australia.
Correspondence: M.G. Mott, Royal Hospital for Sick Children, St Michael's Hill, Bristol, BS2 8BJ, UK.
Received 14 March 1984; accepted 25 June 1984.

Treatment with the combination of radiation therapy to sites of bulk disease and an intensive multi-agent chemotherapy programme initially designed for the treatment of childhood ALL, but with the addition of early cyclophosphamide (the $LSA_2$-$L_2$ protocol), resulted in a dramatic improvement in the prognosis for childhood non-Hodgkin lymphoma (NHL), particularly for those patients who presented with a mediastinal mass (Wollner *et al.*, 1976). In 1977, therefore, six of the centres within the UK Children's Cancer Study Group decided to submit all patients with either T lymphoma or T leukaemia to an intensified variant of our NHL protocol (Mott *et al.*, 1984), the design of which is in some respects similar to the $LSA_2$-$L_2$ regimen.

### Patients and methods

### *The treatment protocol*

The NHL protocol, previously described (Mott *et al.*; 1984 was modified as follows (Figure 1):

1. Induction courses of CHOP were strengthened by the addition of a second injection of vincristine, $1.5\,mg\,m^{-2}$ on day 8, and the continuation of prednisone for 10 instead of 5 days.
2. Treatment courses were given as soon as marrow recovery permitted (usually 12–15 days) rather than at specified 3 week intervals.
3. The two 4-day courses of cytosine arabinoside

© The Macmillan Press Ltd., 1984

**Figure 1** Treatment Schema.

and thioguanine were condensed to form a single 10-day course during which asparaginase was added (CA $100\,mg\,m^{-2}$, 12 hourly × 20, TG $75\,mg\,m^{-2}$ daily × 10, IV Asparaginase 10,000 units $m^{-2}$ alternate days × 5).

4. The three courses of intermediate dose methotrexate given as consolidation therapy were at one week rather than 2 week intervals.
5. All patients received cranial radiation, 17.6 Gy in 8 fractions given over 2 weeks. In addition, patients were randomly assigned to receive or not to receive 15 Gy to the mediastinum concurrently, irrespective of whether there was a mediastinal mass present at diagnosis.
6. Systemic treatment during radiation therapy consisted of a 2-week course of prednisone, $40\,mg\,m^{-2}$ per day and weekly vincristine.

As in the NHL regimen, all patients received a total of 7 doses of intrathecal methotrexate, $10\,mg\,m^2$ (maximum 12 mg) during the induction and consolidation phases.

The maintenance treatment was identical to that for patients with mediastinal primaries on the NHL trial, with oral rather than i.v. methotrexate because of previous cranial radiation, and was continued to complete 2 years of chemotherapy.

*Patient entry*

From November 1977 to July 1983 82 patients with T leukaemia/lymphoma entered this study. There were 65 boys and 17 girls (ratio 4:1) with an age range of 13 months to 15 years. Sixteen had no evidence of bone marrow involvement on either aspirate or trephine biopsy, and 9 had evidence of infiltration of the bone marrow but with <25% blasts. These 25 patients were designated as having had T lymphoma. Fifty-seven patients had more than 25% T lymphoblasts in bone marrow and/or T lymphoblasts in peripheral blood, and were designated as having had T leukaemia; 28 of these 57 had $>100 \times 10^9\,l^{-1}$ blasts in peripheral blood.

There were 27 NHL patients presenting with a mediastinal mass who were treated in the parent NHL trial (Mott *et al.*, 1984). Careful comparison has shown no difference between clinical features at presentation or results of treatment in these patients and the 25 patients treated at 6 centres for T lymphoma in the T cell trial. Therefore for some analyses these two groups of patients with T lymphoma (total 52 patients) were combined.

The methodology used to establish T cell marker status has been described previously (Greaves *et al.*, 1981; Habeshaw, 1980).

### Results

These are shown in Tables I–III and Figures 2–5. There were no significant differences in survival and FFS between the lymphoma patients treated on the NHL and T cell protocols (Table I). Similar results were obtained in the T leukaemia patients with presenting peripheral blasts $<100\times10^9 l^{-1}$, but those with $>100\times10^9 l^{-1}$ blasts did much worse (Figure 3), the difference in survival at 4 years being highly significant ($P=0.003$).

**Figure 2** Overall survival (...) and failure-free (—) survival for all 82 patients in the T cell Trial.

**Figure 3** Failure-free survival for high count T leukaemia (—) *vs* the rest (...).

There were 66 adverse initial events (Table II) recorded for the total of 109 patients with T cell disease (57 patients with T leukaemia and 52 patients with T lymphoma). Forty-one of these 66 adverse events consisted of relapse in bone marrow and/or CNS. Initial bone marrow relapse was more frequent in those with T leukaemia (16/57 *vs* 6/52 for lymphoma) as was CNS relapse (9/57 *vs* 5/52). Death before the achievement of complete remission was more common in T leukaemia (6/57 *vs* 1/52). Patients presenting with T lymphoma had a higher incidence of initial mediastinal relapse (6/52 *vs* 1/57).

Forty-seven of the 52 patients with T lymphoma successfully completed the induction phase of treatment and were randomised to receive or not to receive mediastinal radiation. There was a highly significant difference in FFS between the two groups in favour of the patients randomised to receive radiation (66% *vs* 18%, $P=0.006$) Figure 4. This difference remains significant when all patients with T leukaemia are included (51% *vs* 21%, $P=0.01$). The pattern of adverse events in the two groups of lymphoma patients is shown in Table III.

Five patients were documented to have CNS disease at their initial diagnostic lumbar puncture. Four remain in complete remission 27, 35, 38 and 47 months from diagnosis.

Eleven patients with T leukaemia presented without evidence of a mediastinal mass, and four are surviving 6, 48, 56 and 56 months from diagnosis.

### Discussion

The poor prognosis for T cell disease was firmly established when the advent of cell surface marker

**Table I** T cell lymphoma/leukaemia 4-year survival and failure-free survival

| *Diagnosis* | *Protocol* | *No. of patients* | *% Survival* | *% FF survival* |
|---|---|---|---|---|
| *T lymphoma* | | | | |
| | NHL | 27 | 48 | 39 |
| | T cell | 25 | 54 | 40 |
| | Both | 52 | 49 | 38 |
| *T leukaemia* | | | | |
| WCC $<100\times10^9 l^{-1}$ | T cell | 29 | 53 | 37 |
| WCC $>100\times10^9 l^{-1}$ | T cell | 28 | 20 | 13 |
| All cases | T cell | 57 | 30 | 27 |

**Table II** T cell lymphoma/leukaemia (109 patients). Pattern of 1st adverse event (66)

| | Leukaemia (57) | Lymphoma (52) T cell trial (25) | Lymphoma (52) NHL trial (27) |
|---|---|---|---|
| BM | 16 | 4 | 2 |
| BM+CNS | 2 | 3 | 0 |
| CMS | 9 | 2 | 3 |
| Local | 1 | 1 | 5 |
| Testis | 3 | 1 | 2 |
| Other | 0 | 0 | 2 |
| Death pre CR | 6 | 1 | 0 |
| Death in CR | 1 | 2 | 0 |
| Totals | 38 | 14 | 14 |

**Table III** Pattern of first adverse event in 47 patients with T lymphoma randomised ± mediastinal radiation

| Radiation | + | − |
|---|---|---|
| BM | 1 | 4 |
| BM+CNS | 1 | 2 |
| CNS | 0 | 4 |
| Local | 3 | 2 |
| Testis | 2 | 1 |
| Other | 0 | 1 |
| Death in CR | 1 | 1 |
| Totals | 8 | 15 |

**Figure 4** Failure-free survival for T lymphoma patients randomised ± mediastinal radiation (× RT).

**Figure 5** Overall survival (...) and failure-free (—) survival for 52 patients with T lymphoma.

techniques made it possible to distinguish the disorder from other lymphoid neoplasms of childhood. The present trial had documented an improvement in the prognosis for T lymphoma compared to our recent previous experience (Mathew *et al.*, 1980) and appears to show a highly significant advantage for those patients randomised to receive adjuvant low dose radiation to the mediastinum in addition to chemotherapy. Sixty-six per cent of these patients remain in complete remission with a plateau on the survival curve from 24 months, while patients not given mediastinal radiation have continued to relapse throughout the first two years after completing treatment (Figure 4).

Review of the first adverse events occurring in these patients shows that the major differences between the two arms of the trial are in the frequency of spread to bone marrow and/or CNS (2 *vs* 10, Table III) and in the late occurrence of relapse in the non-radiated patients (Figure 4). The treatment protocol called for randomisation of patients as nearly as possible to the onset of radiation, but this was not feasible in some centres where radiation had to be organised at another hospital. These required longer notice of the need to give mediastinal radiation (randomised) in addition to the non-randomised cranial radiation for all patients. Review of the time to randomisation however shows no apparent difference between the two groups.

There were 3 patients in the group randomised to no mediastinal radiation whose relatively early randomisation before they had completed induction and whose subsequent early adverse events might have prejudiced this arm of treatment. One patient developed clinical symptoms of CNS relapse which was confirmed on CSF cytology before cranial radiation had been administered; the second died in complete remission from sepsis and pancytopaenia associated with intermediate-dose methotrexate administration in sub-optimal circumstances. The third took 20 weeks to complete the induction and consolidation phases of treatment and had widespread disease when he presented for cranial radiation. All might arguably have been excluded as not evaluable on the basis of significant protocol violations. The difference between the two groups however remains significant ($P=0.03$) after their exclusion.

Late relapses up to five years from diagnosis were recorded in a pilot version of this protocol (Levine *et al.*, 1983) and for this reason publication of the results has been delayed to a later stage than in many otherwise comparable studies. A number of those other studies did nevertheless appear to show the establishment of a plateau for survival of T lymphoma after about two years for patients given intensive combination chemotherapy, usually combined with radiation (Duque-Hammershaimb *et al.*, 1983; Anderson *et al.*, 1983; Reihm, 1983).

In our trial where entry was confined to patients with T cell disease, the universal previous finding of a worse prognosis for leukaemic patients with a high initial blast count was again confirmed. The division between favourable and unfavourable prognosis is, however, not set where the arbitrary distinction is drawn between lymphoma and leukaemia (presence of 25% blasts in bone marrow) but is most obvious for T leukaemia patients who present with $>100\times10^{9}\,l^{-1}$ blasts in peripheral blood. Compared with our past experience there has been a shift of the overall survival curve to the right and a corresponding increase in median survival time (24 months for all T leukaemias, 21 months for the high risk group), but late relapses and deaths have brought the plateau of the curve to well below 50%. The median FFS of 16 months for T leukaemia in our study compared favourably with the 13 month figure for a comparable group of patients treated on the POG 7615 study (Pullen *et al.*, 1982).

The relative effectiveness of chemotherapy has enabled us to show that the randomised addition of low dose radiation to the mediastinum can increase the survival rate of some patients, particularly those with relatively localised disease (T lymphoma). This effect was not detected in the companion NHL trial where the FFS curve reached a plateau at a higher and earlier stage, possibly because of the different natural history of B lymphomas (Mott *et al.*, 1984). Review of the failures in the T cell trial suggests that the benefit observed from radiation would not be seen if given in conjunction with more effective systemic chemotherapy.

The clinical features which distinguish patients with T ALL from "Common" ALL (c ALL) are now well established. The disease tends to occur in older children, with a marked predilection for males, and usually presents with a mediastinal mass, a high peripheral blast count and also a relatively high haemoglobin and platelet count. Patients with T ALL have a higher rate of relapse both early and late in the illness than do patients with c ALL. They also have a marked predisposition to involvement of CNS and other sites of extra-medullary disease, such as the testes, both at diagnosis and during the evolution of the disease. Controversy continues about the relative significance of particular prognostic factors such as the initial blast count and the cell phenotype, and the findings are likely to vary between different treatment regimens until more effective treatment is established.

### References

ANDERSON, J.R., WILSON, J.F., JENKIN, D.T. & 8 others. (1983). Childhood non-Hodgkin's lymphoma: The results of a randomised therapeutic trial comparing a 4-drug regimen (COMP) with a 10-drug regimen ($LSA_2$-$L_2$). *N. Engl. J. Med.*, **308**, 559.

CHESSELLS, J.M., HARDISTY, R.M., RAPSON, N.T. & GREAVES, M.F. (1977). Acute lymphoblastic leukaemia in children: Classification and prognosis. *Lancet*, **ii**, 1307.

DUQUE-HAMMERSHAIMB, L., WILLNER, N. & MILLER, D.R. (1983). $LSA_2$-$L_2$ protocol treatment of Stage IV non-Hodgkin's lymphoma in children with partial and extensive bone marrow involvement. *Cancer*, **52**, 39.

GREAVES, M.F. (1981). Analysis of the clinical and biological significance of lymphoid phenotypes in acute leukaemia. *Cancer Res.*, **51**, 4752.

HABESHAW, J.A. (1980). Investigation of non-Hodgkin's lymphoma in children by phenotyping techniques. In *Non-Hodgkin's Lymphomas in Children*, p. 37 (Ed. Graham-Pole.) Masson, USA.

LEVINE, P., BERBERICH, F.R., BURKE, J.R., MOTT, M.G. & WILBUR, J.R. (1983). Lymphoblastic lymphoma: Late relapse in childhood. *Med. Paediatr. Oncol.*, **11**, 33.

MATHEW, P.M., PRANGNELL, D.R., COLE, A.J.L. & 9 others. (1980). Clinical, haematological and radiological features of children presenting with lymphoblastic mediastinal masses. *Med. Paediatr. Oncol.*, **8**, 193.

MOTT, M.G., EDEN, O.B. & PALMER, M.K. (1984). Adjuvant low dose radiation in childhood non-Hodgkin's lymphoma. *Br. J. Cancer*, **50**, **000**.

PULLEN, D.J., SULLIVAN, M.P., FALLETTA, J.M. & 7 others. (1982). Modified $LSA_2$-$L_2$ treatment in 53 children with E-rosette-positive T cell leukaemia: Results and prognostic factors (A Paediatric Oncology Group Study). *Blood,* **60,** 1159.

REIHM, H. (1983). Treatment strategies in childhood acute lymphoblastic leukaemia (ALL). In *Proceedings of the 13th International Congress of Chemotherapy,* Vienna, unpublished.

ROPER, M., CRIST, W.M., METZGAR, R. & 7 others. (1983). Monoclonal antibody characterisation of surface antigens in childhood T cell lymphoid malignancies. *Blood,* **61,** 830.

WATANABE, A., SULLIVAN, M.P., SUTOW, W.W. & WILBUR, J.R. (1973). Undifferentiated lymphoma, non-Burkitt's type. *Am. J. Dis. Child.,* **125,** 57.

WOLLNER, N., BURCHENAL, J.H., LEIBERMAN, P.H. & 3 others. (1976). Non-Hodgkin's lymphoma in children: A comparative study of two modalities of therapy. *Cancer,* **37,** 123.